# Co-Amended Synergistic Interactions between Arbuscular Mycorrhizal Fungi and the Organic Substrate-Induced Cucumber Yield and Fruit Quality Associated with the Regulation of the AM-Fungal Community Structure under Anthropogenic Cultivated Soil

**DOI:** 10.3390/ijms20071539

**Published:** 2019-03-27

**Authors:** Ahmad Ali, Muhammad Imran Ghani, Haiyan Ding, Yang Fan, Zhihui Cheng, Muhammad Iqbal

**Affiliations:** 1 College of Horticulture, Northwest A&F University, Yangling 712100, China; ahmadhort87@nwafu.edu.cn (A.A.); imran_pak@nwsuaf.edu.cn (M.I.G.); woaimama195710@nwsuaf.edu.cn (H.D.); xnyangfan@nwafu.edu.cn (Y.F.); 2 Department of Soil Science & SWC, PMAS-Arid Agriculture University, Rawalpindi-46300, Pakistan; miqbalkhalid@gmail.com

**Keywords:** garlic substrate, mycorrhizal inoculation, Glomus-AM symbiosis, cucumber yield, fruit quality, AM-fungal community composition

## Abstract

Monotonous cucumber double-cropping systems under plastic greenhouse vegetable cultivation (PGVC) previously intensified by long-term anthropogenic activities and manipulative treatments leads to a crop productivity reduction and soil biota disturbances. In this study, the role of the indigenous arbuscular mycorrhizal strain (AM: *Glomus versiforme* L.) and organic substrate (GS: Garlic stalk) application were assessed for plant microbe interaction and crop productivity feedback in a greenhouse (2016–2018) under a cultivated Anthrosol characterized as a replanted degraded soil. We found that repetitively adding AM inocula with organic substrates (GS) improved the cucumber growth and physiology. The useful trait of AM symbiosis with C-amended organic substrates preferentially manifested as increased root colonization, hyphal density proliferation, AM sporulation, root activity, and suppressed Fusarium incidence. The post AM development further prevailed the synergistic interaction, and the co-inoculation effect resulted in an increase in fruit nutrition uptake, seasonal cucumber yield and fruit quality attributes. Illumina MiSeq analysis of the 18S rRNA gene amplicons revealed that the dominant AM genera that are particularly enriched with the *Glomus* taxon may be important ecological drivers associated with plant productivity and fruit quality characteristics. These results suggest that the AM-organic substrate association might be a pragmatic option for use as an economic and efficient biological resource and as a newly-sustainable plant microbe mediator to enhance the regional ecosystem services and plant productivity of the anthropogenic PGVC of this region.

## 1. Introduction

Northern plastic greenhouse vegetable cropping (PGVC) has intensified greatly since 1980 with an area of 2,900,000 ha in 2010 contributing >33% of the total vegetable cultivation across the mainland production [1,2,3]. The conventional management system of this northern PGVC is degrading, due to long-term anthropogenic activities and manipulative treatments. The cultivated soil medium has been associated with single crop repetition, the excessive use of synthetic fertilizers and virulent plant protection measures, which are probably affecting the crop productivity and soil environment [4,5].

Cucumber (*Cucumis sativus* L.) is perceived as an economically important cash crop, and the cucumber double-cropping system is the commonly cultivated planting system in northern China under PGVC conditions [6]. Traditionally mono-cropped cucumber is probably vulnerable to soil-related obstacles, such as soil sickness and soilborne pathogenesis, caused by cucumber autotoxicity, which may reflect the negative plant-soil feedback, due to the imposition of continuous cropping obstacles [7,8]. Previously monotonous cropping systems of more than seven years of continuous cucumber cultivation with conventional management practices are usually considered the major limiting element resulting in a 50% reduction of the total plant biomass, a decline of 31–42% in soil organic matter (SOM) and invasion of the cucumber seedlings with *Fusarium oxysporum* in the northern PGVC of China [9,10]. These cumulative anthropogenic factors occurring over many years drastically disrupted the soil microbial community membership, soil structure instability, and appeared to be the common cause of inferior plant growth, fruit quality deterioration, soilborne disease development and soil biota disturbances [11,12].

Arbuscular mycorrhizal fungi (AMF) are ubiquitous and are the most widespread plant symbionts predominantly found in most natural and anthropogenic ecosystems; they form >80% of the mutualistic associations between terrestrial plant roots and the soil fungal phylum Glomeromycota [13]. Their compatible host-interaction and AM-occurrence level in successive growing seasons with the addition of indigenous mycorrhizal inoculum in degraded soil substrates enables the improvement of the soil structure and reduces ecological risks [14,15].

To offset the completely or partly long-term negative plant-soil feedback, the structure of AMF development and the interactions with other members of the soil community conspicuously contributed to a wide range of functions from plant development to pathogen protection [16]. The useful trait of AM symbiosis is preferentially associated with various organic substrates that respond by promoting effective mycorrhizal inoculation [17], hyphal density proliferation [18] and AM sporulation [19]. Thus, increasing growth promotion, the biocontrol of pathogens [20] and the amelioration of replanted failures [21] indicate that the fungus derives a benefit from the organic substrate [22].

The appearance of most of the AM-Glomeromycota taxa show some level of host preferences and unveiled the diversified role in plant establishment, as well as the assemblage or restructuring of the microbial community composition [21]. In particular, the taxonomic diversity and community structure of the AM-Glomus taxon associated with plant productivity holds an integral part in farming systems [23,24]. Its prevailing symbiosis with terrestrial crops could be paramount to potentially optimizing their role in intensified agricultural ecosystems [25]. Their end uses of synergistic association could be the pivotal ecological driver by inducing plant growth, better physiological development, higher fruit yield, and greater plant productivity under an anthropogenically stressed growth environment [26,27]. However, the effectiveness of particular AMF strains and the progress of AM-infection under the PGVC system have not been vastly exploited.

C-rich crop residues presumably important for intensive production systems could enhance the carbon stock, ample N mineralization processes and improve soil productivity [19,28,29]. Northern PGVC areas have abundant vegetable waste residues, including postharvest garlic materials [30]. However, appropriate vegetable residue management and effective utilization skills for crop production still have not matured among commercial producers. This key obstacle has triggered serious environmental and soil detrimental impacts and thus, severely hinders the sustainable development of the vegetable industry in China.

To address this concern, introducing the diverse allelopathic crop species into the cropping system offers a new crop production dynamic [7,8,11,12,31]. Significantly, the inherent allelopathic potential of garlic crops has attracted wider attention in recent years as a new approach in crop production, representing the most stable and resilient ecological benefits in terms of plant growth regulation, physiological improvement, soil biological modification, and ecological stability [6,32,33]. In crux, garlic stalk residue as a soil amendment alone or in combination with AMF might be a pragmatic option, due to is low cost, efficient biological resource, and environmental friendliness. This could provide broad prospects to lighten and eliminate the continuous cropping based on the PGVC soil obstacle factors. However, the quantification of the permissible amount of garlic residues and its further resource utilization pattern with other biological inputs towards soil-plant productivity feedback have been infrequently reported.

To attenuate this knowledge gap, this study has expanded to explore the more prevalent use of garlic stalk as a soil amendment with AMF to sustain PGVC production. Our preliminary research revealed the confirmatory role of garlic stalk where optimizing a higher amount of garlic stalk in the same replanted Anthrosol induced significant soil quality and plant growth improvement (our unpublished data of spring 2016). Therefore, we pursued the higher concentration of GS from this initial study, and the extent of this study could predict the longer solidity of garlic stalk tested as an organic substrate. In addition, the decomposability of greater amounts of discrete garlic stalks for at least two years of cultivation would verify the allelopathic interference or the allelopathic regulation on the final harvest of the fruit quality traits. Similarly, the potential of the AMF along with crop residue amendments has not been thoroughly studied. Although there is a general belief that a particular fungal strain may not effectively work in the presence of organic substrate, particularly those derived from allelopathic sources, this may not always be true. The aim of this study was to explore the potential of AMF for seasonal cucumber yield and also its ability to confer compatibility towards plant substrate-fungal associations for sustainable cucumber production.

Therefore, the objectives of this study were: (1) To concomitantly estimate the physiological improvement and the associated mycorrhizal development induced by the repeated addition of AMF or organic substrate under anthropogenic PGVC soil; (2) to predict the co-inoculation synergistic interaction for cucumber productivity feedback if the AM examined is adaptable via inoculation induced by applied organic substrates; (3) to elucidate the distribution pattern of AMF communities across the soil samples under single or dual or non-treatment applications, and (4) finally to reveal the GS-AM-mediated changes or assemblage in microbial community composition and diversity associated with subsequent fruit yield and quality traits.

## 2. Results

### 2.1. Effect of the Organic Amendments on Plant Growth Parameters

The continuous addition of either AMF inoculum or garlic stalk (GS) as an organic substrate had a significant impact on the cucumber growth parameters in the anthropogenic soil substrate. Notably, the relative contribution of the sole application of AMF to improve the plant growth variables in the stressed soil substrate was higher than that of the non-amendment or even the garlic stalk application. The co-amendment of AMF and organic substrate (GS) always caused the largest increase in plant growth (Table 1). Plant height and leaf area increased significantly, and the aboveground fresh biomass and belowground dry biomass were approximately one time and two times higher, respectively, in the co-amended soil substrate compared to the continuous non-amended soil. The relative photosynthetic stimulation with organic amendments within the respective non-organic treatments was obviously stronger. A significant improvement in the contents of chlorophyll a, chlorophyll b and total chlorophyll (Chl a + Chl b) was observed in the GS (garlic stalk) +AM-inoculated seedlings, and the corresponding values increased by 29.0%, 58.0% and 35.0%, respectively, compared to the non-inoculated control (Table 2). Cucumber root activity under organic amendments was significantly higher (*p* < 0.05) and was generally not significantly different between the individual applications (Table 2). The AMF applied in the presence of garlic substrate (GS) exhibited significantly higher root activity (64.5%) compared to the control plants.

After the improvements in the synthesis of photosynthetic pigments, the gas exchange variables under amended and non-amended conditions were also significant (Table 3). The net photosynthetic rate (Pn) and stomatal conductance (Gs) of the combined GS+AMF-inoculated seedlings increased significantly by 61.0% and 124.0%, respectively. The transpiration rate (Tr) of the seedlings inoculated with solely GS, AMF and the combined GS+AMF treatments progressively increased, and the Tr rate was 18.9%, 30.2% and 58.8%, respectively, higher compared to the non-amended control (Table 3). The significant difference in the intercellular CO2 concentration (Ci) was observed only at the dual application of GS+AMF compared to the controls.

### 2.2. Effect of Organic Amendments on AMF Development

**Figure 1:**
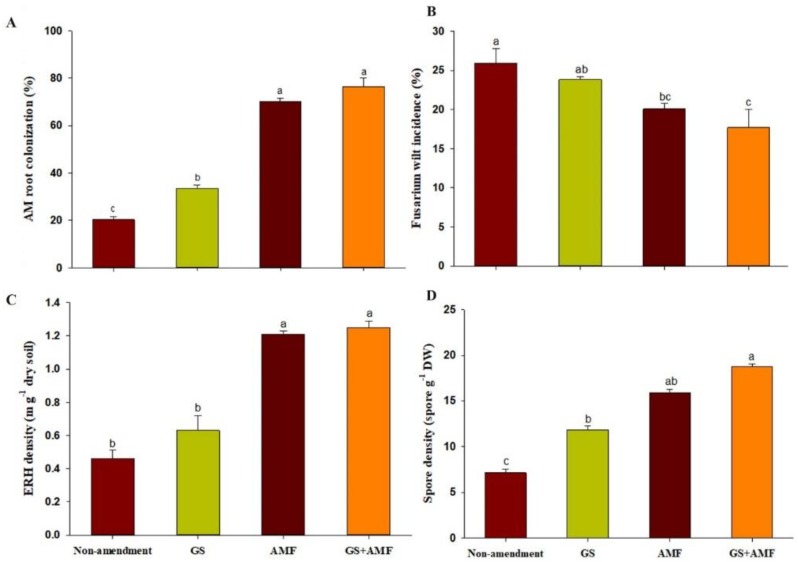
Arbuscular mycorrhizal (AM) root colonization (**A**), fusarium wilt incidence % (**B**), extraradical hyphal (ERH) density (**C**), and spore density (**D**) among treatments. Non-amendment, control; GS, garlic stalk addition; AMF, mycorrhizal Inoculation; GS+AMF, combine garlic stalk and AMF addition. Shared letters above bars denote no significant difference among treatments, as indicated by LSD’s (*p* < 0.05; means ± SE, *n* = 3).

The impact of the application of organic amendments on AMF establishment and associated structural development is remarkable and visible. All the cucumber seedlings were colonized by AMF in the greenhouse. Low levels of indigenous AMF colonization of the roots were detected in the non-inoculated plant plugs (Figure 1A). The exogenous AMF inoculation has substantial potential, due to its 70.2% colonization of cucumber roots. The infection rate of the AM-inoculated cucumber seedling in the presence of the garlic substrate increased by 32.0%, and the colonization strength becomes the highest with a rate of up to 76.0%.

Under conventional cropping, relatively few AMF spores production and hyphal growth were recorded in the cultivated Anthrosol. The inclusion of garlic stalk and AMF resulted in significant increases in spore density and extraradical hyphal (ERH) density when examined after consecutive application four times during each cucumber-growing season (Figure 1C,D). The combination of the GS+AMF resulted in one-fold changes in both densities at the final sampling date (spring of 15 May) compared to continuous non-amendments.

The assayed treatments were also assessed for their biological control potential against cucumber root and stem rot caused by *Fusarium oxysporum* f. sp. radicis-cucumerinum under stress soil conditions, as shown in Figure 1B. The results indicated that a high incidence of Fusarium infection occurred when cucumber plants were subjected to consecutive monoculture for seven years, and the disease symptoms developed to 25.0% until the spring of 2018 under non-amended conditions. The garlic stalk and AMF treatments had an average disease incidence of 23.8% and 20.01%, respectively, and the combined effect of these treatments significantly reduced the incidence of disease by 17.7%, resulting in a control efficacy of 31.7% compared to the control plants.

The results demonstrated that co-inoculation with the AMF and garlic stalks effectively promoted the AMF establishment, enhanced the hyphal growth and sporulation of the AM fungi, and resulted in plant protection in the anthropogenic soil environment.

### 2.3. Effect of Organic Amendments on the Cucumber Yield and Fruit Nutrient Uptake

The seasonal cumulative fruit yields of the greenhouse cucumbers (from autumn 2016 to spring 2018) obtained in the different treatments are summarized in Table 4. Generally, cucumber yields increase in each growing season under all conditions regardless of the application of organic amendments. The individual responses of the AMF and garlic stalk were not significant during the initial two growing seasons of the first year. However, the cucumber yield was significantly different for the next two growing seasons of the second year. The fruit yield was progressively enhanced by 6.0%, 12.9%, 27.7% and 37.8%, respectively, during the first year (autumn 2016 and spring 2017) and second year (autumn 2017 and spring 2018) cultivations after the garlic stalk and AM-inoculation applied together compared to the non-amended plants.

**Figure 2:**
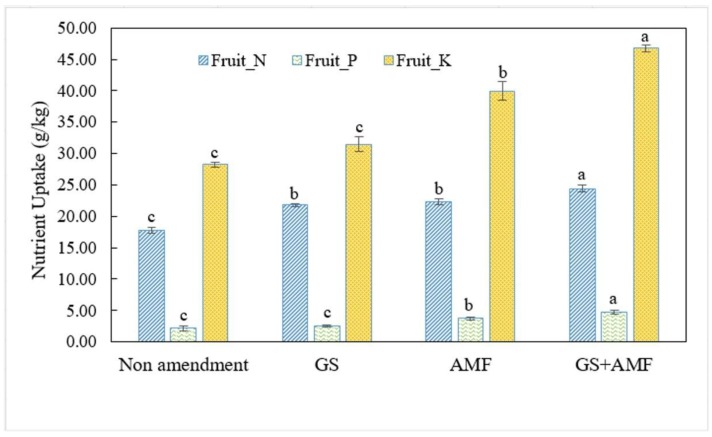
The nutrient uptake analysis in the final harvest of cucumber fruits examined during the spring cultivation of 2018. Different letters indicate the significant differences (*p* < 0.05) by means comparisons (means ± SE, *n* = 3).

The fruit nutrient contents also differed considerably (*p* < 0.05) between the organic amendments and non-amendment under greenhouse conditions. There was a general increase in the N, P and K uptake of the cucumber fruits grown on soil amended with either AMF or garlic stalk compared to the control (Figure 2). The application of AMF and garlic stalk together resulted in the highest fruit N and K uptake by 37.2 and 65.3%, respectively. However, significant differences were not observed in P uptake between the garlic stalk and non-amendment treatment. The treatment with solely AMF contributed significantly to the accumulation of P in cucumber fruit by 72.0% more than control plants.

### 2.4. Effect of Organic Amendments on Fruit Quality Development

The effect of the treatments on fruit quality development was also pronounced, and either soil amended with garlic stalk or AMF inoculation improved the fruit quality traits, as shown in Table 5. Compared to the CK treatment, the dual GS+AMF treatments significantly increased the total soluble solids, soluble sugar, vitamin C, and soluble protein contents of cucumber fruits after two years of continuous cropping (in 2018). The highest total soluble solid and soluble sugar contents (12.2% and 20.7%, respectively) were obtained from the GS+AMF treatment and the lowest from the non-amendment treatment. Discrete applications of GS and AMF also increased the soluble protein content by 1.5% and 3.7%, respectively.

The contents of organic acids decreased slightly by 2.8% with the GS treatment, while the values significantly increased by 7.0% in the GS+AMF treatment compared to the non-amendment. The vitamin C content was significantly induced by organic amendments, and its levels in the cucumber fruit from the GS, AMF and GS+AMF treatments were 13.5%, 23.7% and 49.6% higher than in the CK treatment, respectively. No significant variation in the fruit nitrate accumulation was found between the GS, AMF or non-amendment treatments. However, the GS+AMF treatments caused a significant reduction in the nitrate concentration up to 13% compared to the other treatments.

### 2.5. Effect of AMF Development on Cucumber Fruit Productivity

Cucumber fruit productivity was significantly affected by successful AMF establishment (*p* < 0.05 and *p* < 0.01). Thus, the increases in AM colonization, ERH density, spore density, along with root activity, were positively associated with several fruit quality indices (Table 6). The cucumber yield was positively correlated with AM colonization, ERH density, spore density, and root activity (*r* = 0.950, 0.954, 0.996 and 0.997, respectively) under different organic amendments.

AMF inoculation induced the fruit nutrient uptake, TSS, and SS, due to the addition of organic amendments, and such improvement showed significant positive correlations with AM colonization. The fusarium wilt incidence was significantly and positively correlated with the nitrate content of the fruit but was significantly and negatively correlated with P and K uptake. In addition, these symptoms of diseases showed a similar significant opposite negative correlation for the TSS, SS, and VC contents.

### 2.6. Illumina MiSeq Analysis and the Identification of the AM Fungi

In this study, we identified a total of 533,953 quality-filtered fungal sequences (200–250 bp) and 815 operational taxonomic unit (OTUs) from the 12 libraries across the entire dataset. Among all the sequences retained, the majority of the fungal sequences amplified with AMV4.5NF/AMDGR were detected as AM-*Glomeromycotin* sequences (351,983 sequences, corresponding to 65.9% of the total). However, other fungal sequences classified as non-*Glomeromycota* were also detected as presented in Figure S1. The second and third prevalent groups after the *Glomeromycota* phyla were *Chytridiomycota* (14.1%), and *Ascomycota* (10.2%), respectively. The *Glomeromycota* sequences were extracted for further analyses to identify the phylogenetic distribution of the abundant AM genera across all the soil samples.

**Figure 3:**
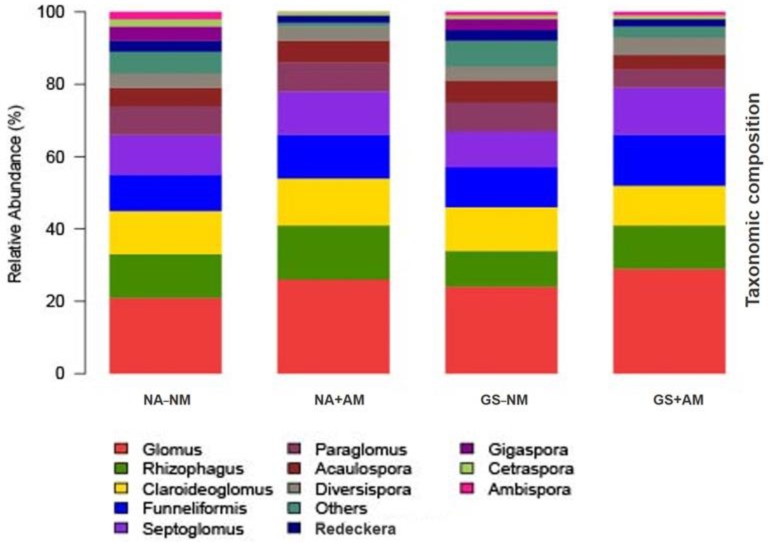
The relative abundance of major identified taxonomic genera of AMF across all treatments. The treatments NA-NM, NA+AM, GS-NM and GS+AM represent the applied soil amendments. Non-amendment and non-mycorrhizal inoculation, non-amended mycorrhizal Inoculation, garlic stalk amended with non-mycorrhizal inoculum and Garlic stalk amended with mycorrhizal inoculum, respectively.

The dominant AMF community composition based on the relative abundance (>1%) of different OTUs affiliated with 11 AMF genera were significantly affected by organic amendments, as shown in Figure 3. The genera consisting of *Glomus*, *Rhizophagus*, *Claroideoglomus*, *Funneliformis*, *Septoglomus*, and *Paraglomus* were the most abundant genera detected among the non-amended and amended soil samples, but their relative levels differed. The relative abundance of the Glomus taxon was 23.8%, 14.3%, and 38.1% higher in the soil samples of the NA+AMF, GS-NM, and AMF+GS, respectively, than the NA-NM samples. Although Rhizophagus was found in all the samples, a greater abundance was detected in the presence of the AMF inoculum (NA+AMF: 15%) than in those of the non-amended non-AM soils (NA-NM: 12%).

Notably, the addition of C-amended garlic stalk as an organic substrate (GS) is important for the proliferation of the AMF community, and its presence or absence caused the significant variation in the community composition of certain AMF taxa. For example, the *Funneliformis* and *Septoglomus* genera were particularly enriched under AMF inoculum soil and showed significant variation after the garlic stalk was applied to the AMF inoculum soil. Their relative abundance associated with the combined application of garlic stalk and AMF improved by 40.0% and 18.2%, respectively, under the GS+AMF treatments compared to the NA-NM treatments. In addition, other taxa, such as *Acaulospora*, *Paraglomus*, *Diversispora*, *Redeckera*, *Cetraspora*, *Gigaspora*, and *Ambispora*, were found in most soils but at relatively low abundances, indicating a good coverage of the *Glomeromycota*.

### 2.7. AMF Community Richness and Diversity

The application of organic amendments significantly affected the OTU richness and phylogenetic diversity of the AMF in this study (Table 7). The highest OTUs (81.44) were recorded under the AMF inoculation treatments of NA+AM, while the lowest OTUs (48.7) were in the controlled treatments of NA-NM. Consistently, the community richness (Chao1 and ACE) and fungal diversity (Shannon index) increased in the soil amended with garlic stalk and mycorrhizal inoculum (GS+AM). The Simpson index was also induced by the applied treatments. However, no significant differences were detected between the NA-NM, NA+AM, GS-NM, and GS+AM soil samples.

**Figure 4:**
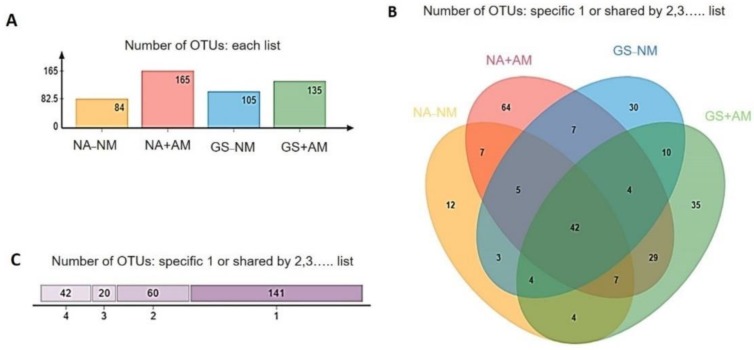
A Venn diagram displaying the degree of overlap or OTUs based co-occurrence of AMF (at the 3% evolutionary distance) among the four treatments. NA-NM: Non-amended Non-mycorrhizal inoculation; NA+AM: Non-amended Mycorrhizal Inoculation; GS-NM: Garlic stalk amended with Non-mycorrhizal inoculum; GS+AM: Garlic stalk amended with mycorrhizal inoculum. Total number of OTUs in each group (**A**), overlapping fashion among all soil samples (**B**), and specific and shared OTUs (**C**) among treatments.

**Figure 5:**
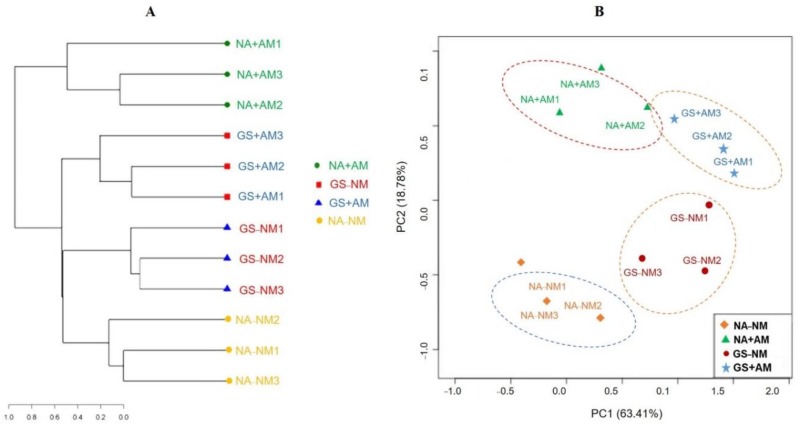
Clustering analysis of AMF communities (**A**), and principal component analysis (**B**) of the different soil samples based on OTU abundance.

A Venn diagram indicated that the soil treated with either mycorrhizal inoculation or garlic stalk showed the distinctive co-occurrence pattern of the OTUs (Figure 4). The NA+AM samples were prominent in unique OTUs, and the unique OTUs accounted for 38.7% of the 165 distinct OTUs observed. Hierarchical clustering analysis revealed that the community structures of the AMF collected from the soils receiving the same treatment clustered together (Figure 5A). The cucumber soil amended with garlic stalk and AMF inoculation was clearly separated from each other. In addition, PCA analysis elucidated the relative number of AMF sequences present in the corresponding genera among all the treatments (Figure 5B). The two principal components accounted for 82.19% (PC1 = 63.41%, PC2 = 18.78%) of the total variances, indicating the great community differences among all the samples.

### 2.8. Interaction between the AMF Community Structure and Cucumber Productivity Factors

**Figure 6:**
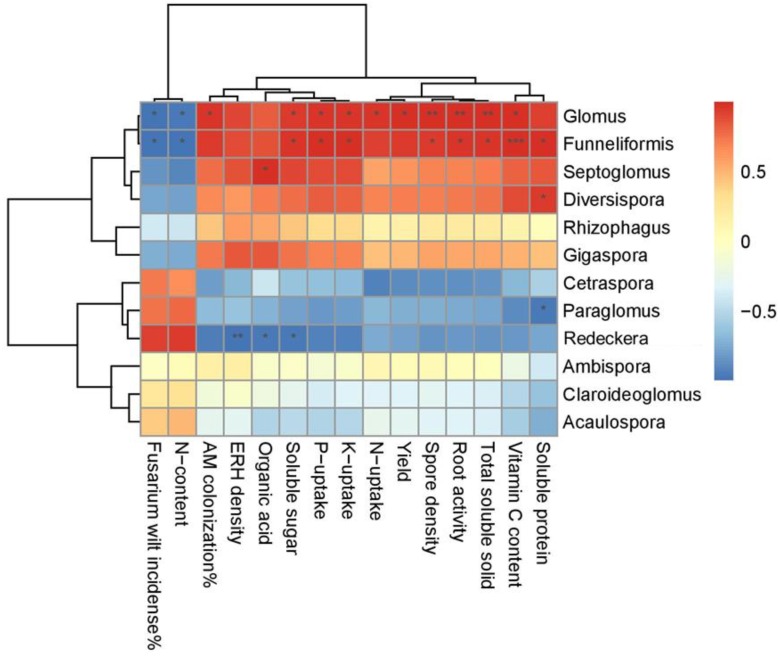
Heat map analysis revealed the relationship between top abundant AMF taxa and cucumber productivity. (Pearson correlation *: *p* < 0.05; **: *p* < 0.01; ***: *p* < 0.001).

The relationships between assigned factors (AMF community and cucumber productivity) were detected using a heat-map correlation analysis, and the intensity of colors suggests a positive or negative relationship between the fruit quality criteria and the abundance of the different fungal classes (Figure 6). The maximum fruit quality development was induced by the AMF-Glomus genera, and their higher abundance significantly (*p* < 0.05) correlated with the yield biomass. Similarly, the abundance of *Funneliformis* is correlated with the fruit TSS (* *p* < 0.05), SS content (* *p* < 0.05), vitamin C (*** *p* < 0.001), soluble protein (* *p* < 0.05), and fruit P and K uptake (* *p* < 0.05), respectively.

In addition, the structure of the AMF development also contributed to the association of the AMF community composition, richness and phylogenetic diversity (revealed by the Shannon’s index) with the garlic stalk amendment and mycorrhizal inoculation variables (Tables S4 and S5). We found that the maximum sporulation, ERH density and root activity were related to the species of Glomus. The improvement of this genus was associated with the cucumber fruit quality criteria. This association implies that the AMF-induced fruit productivity was predominantly attributed to the community composition and diversity of AMF after the organic amendments.

## 3. Discussion

### 3.1. Plant Microbe Interaction for Growth Response Analysis

The experimental data revealed that repeatedly applying the dual inoculants of the GS+AMF during each growing season increased the plant growth and physiological improvement more than singular inoculants. We found that the aboveground biomass, root activity, chlorophyll content, photosynthetic characteristics and nutrient contents in the cucumber seedlings inoculated with AMF under organic amendment were significantly higher than those of the control. Our results were consistent with the previous results that AMF-colonized plants could promote the synthesis of photosynthetic pigments and increase the root expansion area and root activity. This type of colonization also strengthens the absorption and transport of water and other nutrients, and therefore, is involved in the biomass accumulation in plants via the improvement in photosynthetic performance [13,34].

The internal plant metabolism with regards to physiological regulation is the reflex of external plant production indicators, and this study explained that the AM-association with organic substrates in the mycorrhizal cucumber seedlings combined to mediate the physiological functions from plant growth promotion to nutrient accumulation [35,36]. The integral influence of AMF attributed to strengthening the carbon sink, which may trigger photosynthesis in the host plants. AM-colonized roots respond as the strongest sink for carbohydrates because fungi can utilize 20% of the photosynthates produced by their host plants [37]. Consequently, AMF remarkably alters the source-sink relationships by facilitating the exchange of carbohydrates and mineral nutrients. [38,39] hypothesize that AM symbiosis can dramatically increase the gas exchange capacity of the cucumber plants probably by maintaining stomatal opening, reducing stomatal resistance and increasing the transpiration fluxes, and stimulating the photosynthetic traits ultimately causing the improvement of the biomass production of cucumber plants. The results suggest that the GS-AM association is more effective at augmenting plant biomass and crop phenology.

### 3.2. Plant Microbe Interaction for AMF Structural Development and Fusarium Incidence Inhibition

The gradual increase in the limited C-substrate availability and the profession of the pathogenic infection in our PGVC planting system radically hampered the indigenous AM development and restricted mycorrhizal infection, the formation of spores, hyphal growth, and the host-plant root expansion. These AM indicators are the key participants for intensive agroecosystems because of their absence or occurrence, and their particular affiliation with species can determine the mycorrhizal effectiveness and contribute to shaping the community composition [40]. It has also been observed that the root colonization rate is directly associated with the soil spore density [41]. In addition, the results suggested that the AM fungal host plants also secrete chemical factors, which attract and enhance the growth of the developing spore hyphae towards the root system [17].

In this study, the organic substrate (GS) and AM inocula enhanced the cucumber associated AM-colonization, ERH density, spore density, and decreased the infection rate of the Fusarium wilt. The high AM root colonization, sporulation, and ERH density were identified with abundant AM species (Table S4). The development of the *Glomaceae* isolates may be generally attributed as the fastest colonizers of plant roots, and this is consistent with other studies in diversified agroecosystems [26,27]. External nutrient inputs, in particular, the demand for available N and AP, and the plant substrate quality could be factors responsible for the multiplication of AM growth. The host plant roots (and their associated AM fungal traits) articulate the sensitive response to the external P supply. The AM root colonization percentage may decouple from the soil in response to excessive or deficient P supply. Moreover, it was reported that the external hyphae of the AM fungi require a 4–7 times greater N concentration than that of the plant shoots and at least ten times greater than that of the roots [23]. Subsequently, the AM fungi have a substantial N demand and entail large amounts of N for their own growth and structural development [42].

The Glomus hyphae rely on plant-derived carbon and grow prolifically with the appropriate C-allocation through organic material and shift the structure and function of the mycorrhizal communities [43]. The development of the mycorrhizal hyphae in this study could be an integral contribution to the AM-ecosystem services from the plant derived C decomposition and nutrient acquisition from the soil to become available to the microbiota [44]. The added external organic and inorganic inputs were sufficient for the proliferation of AM growth, and this can explain why the AM fungal growth was stimulated by the C-amended garlic substrate [44].

In our cultivation system, it appears that anthropogenic activities greatly reduced fungal development, but were not completely lost following the long continuous cropping spans prior to the establishment of our experiments. We speculate that the fungal taxa were probably present in the system but grew at low undetectable levels. When suitable host plants became established under enriched organic inputs, the AM symbiotic fungi quickly rebounded, increasing root colonization and spore production rates, suggesting that the AM development became more active with exogenous AM+GS amendments over the four seasons of our experiment. Reference [25] also elucidated the responses of AM fungal abundance and community composition simulated by organic amendments found in anthropogenically disturbed environments.

Pre-existing hyphae, germinating spores and infected root fragments are also considered to be the key players in new host establishment. It is also worth noting that the differences in AM development were the greatest within species, and this observation was primarily driven by AM colonization, mycorrhizal hyphal, and sporulation increases of a particular fungus, *Glomeraceae*, known as AM-Glomus spp, indicating the recruiting preferences of the AMF within particular genera, since these results are consistent with those of previous studies [23,45,46].

These consistent results indicated that the new colonization, hyphal growth, spore abundance and host-symbiont interaction of AM-Glomus largely depends on the number of fungal propagules present in the soil, compatible host-plant species, and the types of soil organic additives [14,47]. However, different strains of AM fungi show variable responses, and their mycorrhizal associations may shift from beneficial to detrimental for the plant growth response. This dependency of the heterogenetic response is dependent on the indigenous inoculum potential, new host-range access to compatible symbiont partners, and favorable experimental conditions [21] that have remained largely unexplored in intensive PGVC systems. However, it is clear from this study that the addition of garlic stalk as an organic residue improved the mycorrhizal strength in the degraded environment, directly supporting the hypothesis that the effect of garlic-derived components promoted the structural development of AMF characteristic and contributed in compatible synergistic interaction.

During consecutive trials, we found that cucumber is recognized as a highly mycorrhizal-dependent vegetable crop, which has not only recolonized naturally on this cultivated soil but also can be easily newly colonized by AM fungi (colonized > 75%), and its compatible host interactions are useful to establish of AM associations. In addition, the utilization of garlic residue could ensure the sufficient carbon substrate allocation to the AM fungi. These cumulative conditions probably exert the successful developmental impact in prompt AM synergistic interactions to perform a further role in crop productivity mobilizing belowground microbiota activation [48,49].

A fully developed AM-structure delivers the multiple functions in the association and facilitates the two-way movement of nutrients between the host and mutualistic fungal partner. The symbiotic association more effectively renders the host plant tolerant to various environmental stresses [50]. In this study, we observed the additive effect between the AM fungi and organic substrates (GS) and found a close relationship between the fruit nutritional status and Chl synthesis (Table S3). The increase in the fruit nutrient uptake under the interactive effect of GS+AM was associated with a reduction in Fusarium infection and higher rooting activity (Table S3), suggesting that AM-inoculated seedlings distributed a favorable nutrient profile to the fruit tissue and received extended protection to endure anthropogenic stresses. A possible mechanism of increased N and K uptake and growth in GS+AM inoculated plants may be due to their effects in strengthening the source to sink capacity and resulted in a stimulation of nutrient transfer from the soil to roots and root-fruit tissue [4]. Our results are consistent with those that found that improved P nutrition via mycorrhizal symbiosis (preferably an extension the hyphal length) caused the significant impact on fruit quality and subsequent growth expansion, which are the most important feedback of the synergistic interaction developed in the presence of organic amendments [22].

In sustainable intensive agroecosystems, AM-based bio-inoculants are the most prevalent type of biocontrol agents to ameliorate plant growth and reduce the damage caused by soilborne plant pathogens. We found a co-inoculation antagonistic relationship with a cucumber root pathogen (Fusarium infection) with the AM-inoculum + GS-substrate treatments, and the observed reduction, due to the strength of the applied dual inoculants responded with a more effective induction of rooting structure, increases in root activity, and thus, a pronounced influence on the fruit quality attributes (Table 6, Tables S2 and S3). The pragmatic bio-efficacy of this study could plausibly explain that garlic or garlic-derived potent compounds (flavonoids, sulfur-containing compounds, phenolic, carbohydrates, and antioxidants enzymes) have not only been recognized as bio stimulants [48,49,51] but have also been reported to be strong antimicrobial agents [52,53,54].

The microbial-mediated decomposition of organic materials in the soil can create favorable anaerobic conditions and produce toxic metabolites, which results in the inhibition of soilborne pathogens or the immobilization of their activity [55]. It may be possible that organic or allelopathic compounds released during residue decomposition and their bioactivity in the vicinity of the inoculated host plants may act in three manners; AM compounds that stimulate the plants, a disruption in the AM symbiosis, or action as an inducer for pathogenic fungal resistance [19].

Indeed, it is generally suggested that various garlic tissues/organs (root exudates, garlic stalk, and garlic bulb /root extract) possessed different allelopathic potentials (+tive and -tive) depending on their mode of action, and their specificity has been verified by our previous research findings [11,12,33]. However, plant chemistry, species adaptability, amount, soil type, and pertinent soil edaphic factors are key determinants to predict the derivatives of the organic materials tested [6,7]. By viewing the plant growth response in this study, we hypothesize that the applied garlic substrates comprised of aboveground selective components (only leaves and stalks) are compatible to adopt a particular fungal partner (*Glomus versiforme*), and their putative contribution from growth promotion to plant protection were likely to be due to allelopathic regulation, rather than allelopathic interference. These findings are partially equivalent to other reports in which a synergistic co-inoculation effect through soil treatment improved the yield and fruit quality in a number of vegetable crops in potted studies under greenhouse conditions [56,57].

### 3.3. Plant Microbe Interaction for the AM Community Composition and Cucumber Productivity Feedback

The fungal community identified in this study predominantly assessed from AM-Glomeromycotan sequences using Illumina MiSeq approach (Figure S1) suggests that the AMF have the capacity to interact with others soil microbial communities during litter decomposition [29]. The predominant *Glomus* taxa served as a resilient ecological indicator species with widespread co-occurrence profiling across all the soil samples. These results are consistent with and confirm a previous study that the *Glomus* species were the most abundant in the AMF assemblage, and their enrichment in the soil acts as a mycorrhizal developer that enabled the host plants to develop an effective AM symbiosis [24]. The occurrence of *Glomus* as the dominant members in the AMF assemblage among those of other genera could be influenced by particular factors. The related investigation revealed that the species that belong to the *Glomus* genus could usually emerge with large numbers of spore production and hyphal fragments, which can rapidly colonize and extensively spread onto the AM-infected roots of plants. The influence of these factors may be the reason for the dominance of the Glomus genus in this study [58]. Therefore, these structural improvements assist the profusion and proliferation of *Glomus* genus members in a degraded ecosystem, and the appearance of this developmental phenomenon is also the result of adaptation to the local ecological environment.

Similarly, a remarkable difference in AM fungal diversity was detected after the final spring cultivation of cucumber in 2018 (Table 7). The mycorrhizal seedlings under the GS-substrate significantly induced the Glomus-OTUs richness and diversity (Shannon) that were greatly suppressed in the continuous cropping and NA-NM treatments [18,59]. In addition, the OTU-based AM composition showed that the soil samples from all the treatments at the same sampling site clustered together, indicating similarities in the AMF community composition by hierarchical cluster analysis results (Figure 5A). Variation among the samples from different treatments separated by the PCA analysis indicated that the continuous addition of the GS+AM treatments led to divergences in the microbial community structure and shaped the trajectory of succession in distinctive manners (Figure 5B), which were consistent with these previous studies [60,61].

In this study, the fully developed structure of AM-Glomus was found to be a key ecological driver influencing plant growth and crop productivity. The cucumber yield associated with the Glomus taxon, and therefore, the dominant community composition, significantly increases the seasonal fruit yield, indicating the presence of intrinsic connections of the Glomus-AM symbiosis among biomass production, AM structural improvement and cucumber development. This observation is parallel with other results and implies that the taxonomic associated Glomus community composition and diversity have influenced the crop yield in diversified cropping systems [59]. In addition, this research also confirmed that there are significant relationships between the AMF development, Glomus community structure and fruit quality attributes (Figure 6). The increases in the fruit TSS, SS, OA, VC, and SP were positively associated [62], while the Fusarium suppression and reduction in nitrate concentration were negatively correlated with the enriched Glomus taxon [63]. These interactions were most likely attributable to the fact that the greatest colonization, spore germination and hyphal growth of Glomus species followed by *Funneliformis* can directly exert an impact on fruit productivity traits, which may be stimulated by the favorable mutualism induced by the treatments applied [4,16].

Others causation of the AM community composition and diversity changes, due to the addition of fertilizer supplements, soil biochemical and seasonality impact may contribute to modify the AM fungal community [28]. However, we are uncertain about discriminating the particular influence in this context being beyond the scope of the study. Nevertheless, we found that direct response of growth productivity improvements and community composition changes to these treatments could be an indirect response from the soil modification and successive seasonal impact. Despite the role of these elements, our data suggested that the treatments applied are the sole factor responsible for the shift of the AM fungal community, and the garlic-AM symbiosis may have superseded the influence of these factors on the AM fungal diversity. Thus, derived feedbacks from the AM and organic substrate are the primary implications for local PGVC to understand the composition, diversity, and productivity.

## 4. Materials and Methods

### 4.1. Site Description, Soil and Organic Amendments

Greenhouse experiments were conducted for two years (2016–2018) under a typical eight-year-old commercial plastic tunnel greenhouse (ground area 8 m × 60 m) located at the Horticulture Experimental Station (34°17′ N, 108°04′ E) of the College of Horticulture, Northwest A&F University, China. The experimental topsoil (0–20 cm) was classified as an Orthic Anthrosol (FAO soil taxonomy) with a sandy loam texture, collected from a commercial PGVC structure in which cucumber had been continuously monocropped for seven years (double-cropping cucumber planting system: Spring-March to June, Autumn-August to October). The soil medium used characterized as replanted stressed soil was moderately deficient in organic-C input, and partially suffered from bacterial and soilborne disease prominently with Fusarium wilt [6,12,31].

The raw materials used as an organic substrate were comprised of the aboveground biomass components, i.e., garlic leaves and stalks. The postharvest materials selected were collected from the surrounding areas of the commercial cultivated garlic fields of Yangling, an important intensive region of the northern PGVC, Shaanxi Province. Briefly, the proposed plant substrate components were air-dried, mechanically smashed, homogenized into powder (sieve > 0.5 to 1 mm) and stored at 4 °C prior to use. The dehydrated and finely crushed garlic stalk/residues were thoroughly mixed and incorporated as a soil amendment in each growing period. The selected analytical characteristics of the organic residue and replanted soil were determined prior to treatment implementation, and the results are shown in Table S1.

### 4.2. Source of Inoculum and Maize-Trap Culture of AMF Propagation

The indigenous AM fungus used in the study was a strain of Glomus (*Glomus versiforme* L.), which was provided by the College of Horticulture, Northwest A&F University, Yangling, China. The reference AM inoculum was propagated using maize (*Zea mays* L.) trap culture media, and the potted mixture as the substrate was used to multiply the native AM inoculum to obtain the sufficient quantity of inoculum potential required for the experiments. The trapped plants were cultured for four successive propagation cycles, four months each the under axenic conditions of a controlled greenhouse (25/16 °C day/night, 70–75% of relative humidity, 16/8 light/dark photoperiod with 750 μmol m^−2^ s^−1^ photosynthetic photon flux density). Finally, the harvested inoculum of the pot mixture, containing spores, hyphae, and segmented mycorrhizal roots, were air dried, sieved (2 mm), and stored at 4 °C prior to use. Autoclaved substrate represented the non-mycorrhizal inocula (containing growth medium and root) and was also prepared to use for non-amended and non-AMF cucumber plants.

### 4.3. Experimental Set-Up, Plant Materials and Treatments Applications

Investigations were conducted during four successive growing seasons (two years) of spring and autumn cultivation of the cucumber crops (Autumn-2016; Spring-2017; Autumn-2017; Spring-2018) under a plastic tunnel greenhouse. The span of the autumn cultivation started from 10 August –20 November, while the spring cultivation span started from 5 March–21 June each year, respectively. The experimental setup for each growing season was similar, including a complete randomized design with three replicates of four treatments. A total of 120 plastic pots (15 cm × 15 cm × 15 cm of each) containing 8 kg of replanted soil was prepared, and ten pots for each treatment were placed in a plastic tunnel. Four treatments were assigned in this study, including soil amendment with garlic stalk as the organic substrate (GS), arbuscular mycorrhizal fungi inoculation (AMF), a combination of organic substrate and arbuscular mycorrhizal inoculation (GS+AMF) and non-amendment with non-AMF inoculation as the control (CK).

The cucumber seeds (*Cucumis sativus* L. cv. Jinglu No. 3) were surface sterilized with hypochlorite solution (1%) and sown in germination trays. These trays were maintained at 28 °C in the dark in a plant growth chamber for one month. The uniformly sized seedlings from these trays were transplanted into each pot at the 4th leaf stage maintaining one cucumber plant per pot. Before transplantation, 15 g of organic fertilizer (“Peng-Di-Xin”, manure substitute, Henan, China), containing 30% organic matter, 4% N+P_2_O_5_+K_2_O, 20% humic acid, 2% trace elements, and 5% organic sylvite) and 15 g of compound fertilizer (N-P_2_O_5_-K_2_O: 18:18:18) were added as a basal fertilizer for each growing season according to local recommendation.

The organic substrate as the soil amendment alone or in combination was incorporated at the rate of 5 g/100 g dry soil, while 25 g of propagated mycorrhizal inoculum (approximately 2200 infective propagules/g trap soil) as the AMF treatment alone or dual application was used in this study. The prepared inoculum was placed adjacent to the AM-treated roots (5 cm below) of the seedlings and in the vicinity of the organic substrate soil during each growing season. Non-AMF plants serving as the control received the same weight of mycorrhizae-free substrate (autoclaved inocula) without organic amendment.

### 4.4. Soil and Plant Sampling

In July 2018, soil samples were collected from each pot and mixed as a composite sample after cultivating the cucumbers for four growing seasons with the applied treatments. A total of 12 soil samples (4 treatments × three replicates × one sampling time) were obtained and immediately transported to the laboratory on ice. The soil samples were divided into two parts after manual inspection, sorted (<2 mm) and divided into two portions. One part was stored at −80 °C for molecular MiSeq analysis and the AM fungal hypha measurements; the other was air-dried to analyze the AMF fungal spore density and soil physicochemical characteristics. Similarly, the plant morpho-physiological observations and fruit quality analysis of the cucumber were also performed in triplicate for each treatment after the final harvest of the cucumber in July 2018.

### 4.5. AM Root Colonization, Extraradical Hyphal (ERH) Density, and Spore Density

After four seasons of treatment application, a portion of the experimental soil was evaluated for indigenous AMF occurrence in the soil used to cultivate cucumbers. To assess the percentage of mycorrhizal infection of the AM-inoculated and non AM-inoculated cucumber roots, a fraction of the fine root samples (0.5 g) was placed in 10% KOH (*w/v*) at 90 °C for 20 min and stained with 0.05% (*w/v*) trypan blue in lactoglycerol as described by [64]. The clear extent of the colonization in the stained root segments (1 cm in length) was examined using light microscopy. The amount of the mycorrhizal colonization infection was calculated as the percentage of mycorrhizal infected root lengths against the total stained root lengths.

To estimate the ERH density, 4.0 g soil from each sample was extracted using the membrane filter method described by [65] and separated into AM and non-AM fungal hyphae based on their morphology and staining color examined at 200× magnification [66]. The hyphal length was estimated using the grid-line intercept method.

The spore density was calculated using the wet sieving and decanting method described by [67]. The AM spores were extracted from 20.0 g air-dried soil of each sample. The soil was placed in a 32-μm-mesh sieve and vigorously washed with deionized water. The spores were isolated from the larger soil particles and extracted with a 60% sucrose solution after 3 min of centrifugation. The cleaned, bright, and apparently viable spores were counted on a gridded Petri dish, examined microscopically under 50× magnification, and the fungal spores were identified to the species-morphotype and quantified as described by [68].

### 4.6. Plant Growth Observations

The cucumber leaf area was analyzed using a portable leaf area meter (AM-350, England). The plant fresh and dry biomass of the aboveground and belowground tissues were measured using an electronic precision balance (0.001 g). Our experimental soil typically exhibited continuous stressed and Fusarium invaded soil. Therefore, we monitored the Fusarium wilt observations, including foliage chlorosis, leaf dropping and necrosis until the harvest of each growing season. The average disease incidence until the final plantation was expressed as the percentage of infected plants per total number of plants.

A LI-6400XT portable photosynthetic system (LI-COR^®^ Biosciences 6400XT, Lincoln, NE, USA) was utilized to measure the net photosynthetic rate (Pn), intracellular CO_2_ rate (Ci), transpiration rate (Tr), and stomatal conductance (gs). All the gas exchange measurements were recorded at the third fully expanded cucumber leaves from the apex, and the conditions were maintained under a CO2 level, PPFD and air temperature at 400 µmol mol^−1^, 1000 µmol m^−2^ s^−1^, and 25 °C, respectively.

To determine the root activity, 0.5 g of fresh root tissues were homogenized in 10 mL 0.5 mM phosphate buffer solution containing 0.4% (*w/v*) triphenyl tetrazolium chloride (TTC) as described by [69]. The spectrophotometric absorbance of the obtained extract was measured at 485 nm. The root activity was calculated using the following equation: Root activity (µg.g^−1^.FW h^−1^) = amount of TTC reduction (µg)/fresh root weight (g) × time (h).

The photosynthetic pigments were extracted from the fourth freshly developed leaves (0.5 g) in 80% acetone (*v/v*) and incubated in the dark for 24 h at 4 °C. The absorbance reading of the crude extract of the supernatant of chlorophyll a and chlorophyll b was recorded colorimetrically [70] at 663 and 645 nm using a spectrophotometer (UV-3802, UNICO, NJ, USA).

### 4.7. Nutrient Acquisition Analysis, Fruit Quality Evaluation and Fruit Yield

After the final harvest of the cucumber in July 2018, three randomly selected plant samples per replicate were divided into different plant parts (shoots, roots and fruits), rinsed with distilled water and oven-dried separately at 70 °C for 72 h. The dried samples of the fruits were ground and homogenized into powder before nutrient analysis. To analyze the fruit N, P and K, the samples (0.3 g dry mass) were pre-digested with a mixture of 3 mL con. HNO_3_ (62%) and 3 mL of H_2_O_2_ (30%) as described previously [31]. The N content was determined using a semi-micro Kjeldahl apparatus. The phosphorus (P) content was assessed spectrophotometrically, and the potassium (K) content was measured using an atomic absorption spectrophotometer.

For the evaluation of the fruit quality indices, the nitrate contents were extracted using boiling water and evaluated using UV-spectrophotometry [71]. The soluble solid contents (°Brix) in the cucumber fruits were recorded using a portable refractometer (WYT-4, CANY, Shanghai, China). The organic acid contents were measured using direct NaOH titration, and the soluble sugar contents were determined using the anthrone colorimetric method. The fruit soluble protein content was recorded using a Coomassie brilliant blue G-250 solution, and the vitamin C content was measured using a 2,6-dichlorophenol indophenols titration method previously described [71].

The cucumber yield was recorded during four cropping seasons starting on 8 September 2016, 17 April 2017, 11 September 2017, and 13 April 2018, respectively. Average commercial fruits graded as marketable fruits (2.5–3.0 cm in diameter and 25–30 cm long) were picked following conventional harvesting practices and weighed from six plants in each replicated treatment. After the final fruit harvest of each crop, the cucumber vines were removed from the greenhouse, and the pots were covered with plastic film to minimize the risk of fungal disease.

### 4.8. Molecular Profiling of the Fungal Communities Using Illumina Analysis

To further examine the cucumber-associated AMF community composition and diversity after four times of amended and un-amended conditions, we employed Illumina MiSeq sequence analysis approach. There were 12 soil samples (three for each treatment) of the AM plants adhering with the mycorrhizal colonized roots, organic amended plants and non-AM mycorrhizal plants that were collected after the final cucumber harvest in July 2018, stored at −80 °C and subjected to DNA extraction.

### 4.9. DNA Extraction and Genomic Amplification

The total DNA extraction from 0.5 g frozen soil samples was conducted using a PowerSoil^®^ DNA Isolation Kit (MoBio Lab, Inc., Carlsbad, CA, USA) according to the manufacturer’s instructions. The qualitative and quantitative assay of the extracted DNA was performed using 1.0 % agarose gel electrophoresis and ND-2000 UV–Vis spectroscopic analysis (NanoDrop Technologies, Wilmington, DE, USA) and stored at −20 °C for subsequent PCR reactions.

The 18S rRNA gene was amplified using a thermocycler PCR system (GeneAmp 9700, ABI, Foster, CA, USA) with the primer set of AMV4.5NF (5′-AAGCTCGTAGTTGAATTTCG-3′)/AMDGR (5′-CCCAACTATCCCTATTAATCAT-3′). These primers are vigorous and specific to accurately express the indigenous AMF communities on the Illumina MiSeq platform [72,73]. The initial PCR amplification was performed in triplicate in a reaction mixture (25 μL) containing 0.4 μL of 5 × FastPfu Buffer (Sangon Biotech, Shanghai, China), 2 μL of 2.5 mM dNTPs, 0.8 μL of each primer (5 μM), 0.4 μL of Fast Pfu Polymerase (Takara), 0.2 μL bovine serum albumin (Takara) and 10 ng of template DNA. The final PCR reaction proceeded with the following cycling conditions: Three minutes of initial denaturation at 94 °C, followed by 30 cycles at 94 °C for 30 s, 30 s for primer annealing at 55 °C, 45 s for elongation at 72 °C, followed by a final extension period at 72 °C for 10 min. The resulting PCR products were pooled and visualized on a 2.0% agarose gel, purified further using an AxyPrep™ DNA Gel Extraction Kit (Axygen Biosciences, Union City, CA, USA) and quantified using QuantiFluor™-ST (Promega, Madison, WI, USA) according to the manufacturer’s instructions. Amplicon sequencing was processed using the Illumina MiSeq platforms at Genedenovo Technology Co., Ltd. Inc. (Guangzhou, China).

### 4.10. Processing of Sequence Data

The Illumina sequencing data were demultiplexed and analyzed using the combination of the software Quantitative Insights Into Microbial Ecology (QIIME: V1.9.1, http://qiime.org/index.html), UPARSE pipeline (v7.0.1001, http://drive5.com/uparse/) and R package (v3.2.2). The raw reads data were quality-filtered using the QIIME toolkit [74]. After optimizing the sequences, only unambiguous sequences identified as AMF were clustered into operational taxonomic units (OTUs) at 97% similarity using the UPARSE pipeline [75]. The phylogenetic affiliation of the most abundant sequence from each OTU was designated as a representative sequence for OTUs that reached the 97% similarity level. The taxonomic assignments of each 18S rRNA gene sequence were analyzed using the RDP classifier (http://rdp.cme.msu.edu/) against the SILVA (SSU123). Different OTUs were further assigned for alpha diversity (Shannon and Simpson), richness (Ace and Chao), and Venn diagram analyses using MOTHUR32. All the representative sequences were checked against the MaarjAM AMF database.

### 4.11. Statistical Analysis

Data corresponding to the plant growth and metagenomic profiling for the AMF community (mean ± SE, *n* = 3) were analyzed by a one-way analysis of variance (ANOVA) for a completely randomized design using SPSS 13.0 (SPSS, Inc., Chicago, IL, USA). The significant differences between the treatments were compared using the least significant differences LSD test (*p* < 0.05).

Cluster analysis was conducted using R v. 3.2.2 software to evaluate the microbial community composition based on a similarity matrix generated using the Bray-Curtis method. The PCA analysis was used to evaluate the overall differences in microbial community composition among different samples (based on OTU abundance). In addition, to determine the impact of the AMF community composition and diversity on cucumber productivity, a heat map was constructed to show the correspondent association among the parameters using Pearson’s correlation coefficients.

## 5. Conclusions and Perspectives

In conclusion, this study corroborates the first outlook that the AM-Glomus taxon has a stronger ecological niche in our anthropogenic PGVC systems. The potential role of indigenous organic inputs attributed to improving the structural development of native mycorrhiza via typical mutualistic symbiosis with compatible host plants. The GS-AM-mediated community composition and diversity primarily derived from *Glomus* mycorrhizae significantly contributed to cucumber yield and fruit quality attributes. Indigenously applied biological inputs are effective to trigger the compatible plant microbe interaction for ecological restoration of the anthropogenic PGVC of this region. The potted trials provided further support for the causal nexus between the organic-AM association in the alleviation of soil continuous cropping obstacles particularly under the condition of C limitation with a naturally low abundance of AMF spores in the PGVC Anthrosol. Such mutualistic results re-affirm the physiological, as well as the ecological significance, of the joint management of mycorrhizae, and the organic amendments suggest avenues for a sustainable intensive PGVC in China. Based on these observations, we recommend future AMF effect research to provide an emphasis in exploring the more compatible AM-symbiosis from other mycorrhizal vegetable families by utilizing locally abundant low-input technology (organic amendments). Their synergistic association must be advocated for crop productivity improvement in intensively degraded ecosystems.

## Figures and Tables

**Table 1:** The plant growth attributes of greenhouse cucumber under various treatments examined during the spring cultivation of 2018.

Treatments	Plant Height (cm)	Leaf Area (cm^−2^)	Shoot FW (g/plant)	Root DW (g/plant)	Fruit Length (cm)
GS+AMF	115.38 ± 1.40 a	217.82 ± 1.38 a	128.3 ± 0.49 a	2.14 ± 0.29 a	34.12 ± 0.84 a
AMF	96.59 ± 1.6 b	194.99 ± 2.0 b	117.97 ± 1.57 b	1.85 ± 0.12 a	31.26 ± 2.17 ab
GS	100.98 ± 2.6 b	192.77 ± 2.25 b	110.69 ± 2.17 c	2.08 ± 0.28 a	30.08 ± 0.68 b
Non-amendment	87.47 ± 1.76 c	182.88 ± 2.1 c	85.84 ± 1.74 d	0.84 ± 0.14 b	28.93 ± 1.36 b

Values within a column followed by the same letter(s) are not significantly different at *p* < 0.05, based on one-way analysis of variance with LSD test. Non-amendment: (Control); GS: (Garlic stalk only); AMF (Mycorrhizal inoculum only); GS+AMF (garlic stalk and arbuscular mycorrhizal fungi inoculation).

**Table 2:** Effect of garlic stalk and AMF on photosynthetic pigments and root activity of spring cucumber of 2018.

Treatments	Chl a (mg/g FW)	Chl b (mg/g FW)	Total Chl (mg/g FW)	Root Activity (μg.g^−1^.FW h^−1^)
GS+AMF	3.42 ± 0.25 a	1.11 ± 0.34 a	4.54 ± 0.63 a	64.47 ± 0.29 a
AMF	3.22 ± 0.12 ab	0.96 ± 0.18 a	4.18 ± 0.21 a	56.24 ± 1.82 b
GS	3.1 ± 0.18 ab	0.91 ± 0.04 b	4.02 ± 0.17 a	51.47 ± 2.42 b
Non-amendment	2.65 ± 0.16 b	0.7 ± 0.19 c	3.35 ± 0.29 b	39.47 ± 0.96 c

Values within a column followed by the same letter(s) are not significantly different at *p* < 0.05, based on one-way analysis of variance with LSD test.

**Table 3:** Effect of garlic stalk and AMF on photosynthesis and leaf gas exchange measurements of cucumber in spring-2018.

Treatments	Pn Rate (μmol CO_2_ m^−2^ s^−1^)	Gs (mol H_2_O m^−2^ s^−1^)	Ci (μmol CO^2^ mol^−1^)	Tr (mmol H_2_O m^−2^ s^−1^)
GS+AMF	25.26 ± 1.41 a	0.65 ± 0.14 a	367.61 ± 2.54 c	6.94 ± 0.99 a
AMF	23.26 ± 2.78 ab	0.48 ± 0.5 ab	351.93 ± 1.92 b	5.69 ± 0.38 ab
GS	22.84 ± 0.62 b	0.45 ± 0.10 ab	343.13 ± 2.87 ab	5.2 ± 0.46 ab
Non-amendment	15.66 ± 0.44 c	0.29 ± 0.03 b	313.95 ± 1.08 a	4.37 ± 0.41 b

Mean values (means ± SE, *n* = 3) within columns followed by different letters are significantly different using the LSD test (*p* < 0.05). Net photosynthetic assimilation (Pn); Stomatal conductance (Gs); Intercellular CO2 concentration (Ci) and transpiration rate (Tr) in fully expanded cucumber leaves in response to organic amendments.

**Table 4:** The seasonal greenhouse cucumber yield (g/plant) in two years under plastic greenhouse vegetable cultivation (PGVC) amended and non-amended treatments.

Treatments	First Year Cultivation		Second Year Cultivation		Average Yield
	Autumn-2016	Spring-2017	Autumn-2017	Spring-2018	
GS+AMF	971.51 ± 3.50 a	1067.06 ± 2.5 a	1227.9 ± 1.47 a	1338.62 ± 3.5 a	1151.27 ± 2.74 a
AMF	963.51 ± 2.50 a	1026.5 ± 3.6 b	1132.1 ± 5.14 b	1207.91 ± 1.71 b	1082.50 ± 3.23 b
GS	968.06 ± 4.48 a	1011.39 ± 1.75 b	1071.6 ± 2.61 c	1184.58 ± 2.64 c	1058.90 ± 2.87 b
Non-amendment	916.23 ± 1.68 b	945.12 ± 3.19 c	961.79 ± 1.83 d	971.19 ± 3.16 d	948.58 ± 2.46 c

Values (means ± SE, *n* = 6) within a column followed by the same letter(s) are not significantly different at *p* < 0.05, based on one-way analysis of variance with LSD test. CK: (non-Mycorrhiza inoculum); GS: (garlic stalk only); AMF (Mycorrhizal inoculum only); GS+AMF (garlic stalk and AMF inoculation).

**Table 5:** Cucumber fruit quality indices in sprig of 2018 in the amended and non-amended treatments under PGVC soil.

Treatments	Total Soluble Solid (^0^Brix %)	Soluble Sugar (mg/g)	Organic Acid (g/kg)	Vitamin C (mg/kg)	Soluble Protein (mg/g)	Nitrate Concentration (mg/kg)
GS+AMF	4.5 ±0.26 a	37.83 ± 0.42 a	1.51 ± 0.14 a	107.99 ± 0.87 a	2.10 ± 0.09 a	68.06 ± 8.40 b
AMF	4.33 ± 0.21 b	35.77 ± 0.29 b	1.48 ± 0.04 ab	89.31 ± 0.92 b	1.95 ± 0.04 b	71.51 ± 4.11 ab
GS	4.23 ± 0.11 b	32.05 ± 0.27 c	1.37 ± 0.03 c	81.98 ± 1.62 c	1.91 ± 0.03 c	76.96 ± 6.20 a
Non-amendment	4.01 ± 0.33 b	31.35 ± 1.05 c	1.40 ± 0.08 bc	72.20 ± 0.85 d	1.88 ± 0.01c	78.40 ± 7.30 a

Values (means ± SE, *n* = 3). Mean values within columns followed by different letters are significantly different using the LSD test (*p* < 0.05).

**Table 6:** Correlation analysis of AMF development with cucumber fruit productivity.

AM-Indices	TSS	SS	OA	VC	SP	N-NO ^3−^	N-Uptake	P-Uptake	K-Uptake	Yield
AM colonization	0.972 *	0.965 *	0.867	0.931	0.855	−0.969 *	0.978 *	0.963 *	0.970 *	0.950 *
Fusarium wilt	−0.970 *	−0.990 **	−0.910	−0.963 *	−0.913	0.994 **	−0.917	−0.992 **	−0.995 **	−0.968 *
ERH density	0.898	0.975 *	0.943	0.876	0.812	−0.971 *	0.832	0.952 *	0.959 *	0.954 *
Spore density	0.998 **	0.919	0.768	0.955 *	0.880	−0.931	0.990 *	0.940	0.944	0.996 **
Root activity	0.999 **	0.919	0.766	0.962 *	0.893	−0.932	0.990 **	0.942	0.946	0.997 **

* Correlation is significant at the 0.05 level (2-tailed). ** Correlation is significant at the 0.01 level (2-tailed). TSS: Total soluble solid; SS: Soluble sugar; OA: Organic acid; VC: Vitamin C; SP: Soluble protein; N-NO^3-^: Nitrate content.

**Table 7:** Comparison of the estimated OTUs richness and diversity indices of AMF clustering at 97% identity, as obtained from the Illumina MiSeq sequencing under different soil samples.

Treatment Code	OTUs	ACE	Chao	Shannon	Simpson
NA-NM	48.7 ± 6.62 c	55.22 ± 6.23 c	57.33 ± 4.06 c	0.95 ± 0.16 b	0.11 ± 0.5 a
NA+AM	81.44 ± 7.61a	84.44 ± 5.00 a	89.73 ± 6.64 a	2.14 ± 0.32 a	0.21 ± 0.6 a
GS-NM	59.7 ± 8.17bc	61.18 ± 1.23 bc	64.21 ± 7.21 c	1.12 ± 0.15 a	0.18 ± 0.4 a
GS+AM	73.5 ± 5.78 b	75.64 ± 4.87 ab	77.55 ± 8.59 b	2.15 ± 0.24 a	0.21 ± 0.3 a

Average of three replicates with standard error (*n* = 3, mean ± SE) of each treatment. NA-NM: Non-amended Non-mycorrhizal inoculation; NA+AM: Non-amended Mycorrhizal Inoculation; GS-NM: Garlic stalk amended with non-mycorrhizal inoculum; GS+AM: Garlic stalk amended with mycorrhizal inoculum.

## Supplementary Materials

The following are available online at https://www.mdpi.com/1422-0067/20/7/1539/s1.
