# Supplementary material for: Co-Amended Synergistic Interactions between Arbuscular Mycorrhizal Fungi and the Organic Substrate-Induced Cucumber Yield and Fruit Quality Associated with the Regulation of the AM-Fungal Community Structure under Anthropogenic Cultivated Soil
Source: Int J Mol Sci. 2019 Mar 27;20(7):1539. doi: 10.3390/ijms20071539 (PMC6479614; doi:10.3390/ijms20071539)
Supplement: Supplementary file 1 [file ijms-20-01539-s001.pdf]

Co-amended synergistic interactions between AMF and the organic substrate-induced cucumber yield and fruit quality associated with the regulation of the AM-fungal community structure under anthropogenic PGVC soil.

Ahmad Ali [1], Muhammad Imran Ghani [1], Haiyan Ding [1], Zhihui Chenga [1,*], Muhammad Iqbal [2]

1 College of Horticulture, Northwest A&F University, Yangling, Shaanxi 712100, China.

2 Department of Soil Science & SWC, PMAS-Arid Agriculture University, Rawalpindi-46300, Pakistan.

*Corresponding Author: Zhihui Cheng

E-mail: chengzh@nwsuaf.edu.cn
Phone & fax number: +86 29 87082613

Supporting Information

6Tables

1 Figure

Table S1: Basic characteristics of replanted anthrosols and garlic stalk before experiment

Parameters	Replanted soil	Garlic substrate
pH (1:5 soil: water)	7.75	7.25
EC (μs·cm^{-1})	582	671
Organic C (g·kg^{-1})	6.59	411.39
Total N (g·kg^{-1})	1.438	8.43
C:N	9.45	45
Total P (g·kg^{-1})	0.93	18.74
Total K (g·kg^{-1})	7.15	10.27
Available N (mg·kg^{-1})	53.65	-
Available P (mg·kg^{-1})	59.41	-
Available K (mg·kg^{-1})	305.91	-

Table S2: Correlation analysis between different growth indices

Pearson's correlation between AMF development indices				
	Fusarium wilt incidence %	ERH density	Spore density	Root activity
AMF colonization %	-0.990*	0.973*	0.975*	0.970*
Fusarium wilt incidence %	1	0.782	-0.964*	-0.963*
ERH density		1	0.899	0.891
Spore density			1	0.999**
Root activity				1

*Correlation is significant at the 0.05 level (2-tailed).
**Correlation is significant at the 0.01 level (2-tailed).

Table-S3: Correlation analysis of plant growth and biomass with photosynthesis and leaf gas exchange attributes

	N-uptake	P-uptake	K-uptake	Yield	Plant height	Leaf area	SFW	RDW
Chl a	.997**	.958*	.977*	.996**	.892	.884	1.000**	.923
Fusarium%	-.917	-.992**	-.995**	-.938	-.828	-.908	-.934	-.723
Chl b	.995**	.926	.928	.999**	.935	.935	.990**	.894
Chl a+b	.999**	.907	.972*	.999**	.911	.904	.998**	.917
Root activity	.990**	.942	.946	.997**	.915	.928	.992**	.876
Pn rate	.986*	.807	.815	.973*	.862	.814	.984*	.974*
Gs rate	.976*	.937	.936	.986*	.966*	.972*	.965*	.853
Ci rate	.997**	.917	.921	.998**	.905	.905	.998**	.907
Tr rate	.946	.974*	.972*	.965*	.942	.982*	.941	.777

**. Correlation is significant at the 0.01 level (2-tailed).

*. Correlation is significant at the 0.05 level (2-tailed).

Table-S4: Correlation analysis of abundant AMF taxa with AMF development

**AMF taxa**	**AM colonization (%)**	**Fusarium wilt incidence (%)**	**ERH density**	**Spore density**	**Root activity**
Glomus	0.975*	-0.983*	0.965*	0.991**	0.993**
Rhizophagous	0.435	-0.386	0.592	0.238	0.210
Claroideoglomus	-0.155	0.261	-0.055	-0.289	-0.322
Funneliformis	0.940	-0.972*	0.894	0.954*	0.961*
Septoglomus	0.779	-0.848	0.864	0.685	0.689
Paraglomus	-0.664	0.754	-0.617	-0.716	-0.738
Acaulospora	-0.273	0.407	-0.293	-0.295	-0.322
Diversispora	0.664	-0.754	0.617	0.716	0.738
Redeckera	-0.932	0.937	-0.991**	-0.832	-0.823
Cetraspora	-0.806	0.729	-0.674	-0.867	-0.858
Gigaspora	0.732	-0.716	0.859	0.564	0.545
Ambispora	0.157	-0.024	0.179	0.074	0.041

*. Correlation is significant at the 0.05 level (2-tailed).
**. Correlation is significant at the 0.01 level (2-tailed).

Table S5: Pearson correlations (r) between AMF alpha-diversity and cucumber development

	AMF alpha-diversity indices				
	OTUs	ACE	Chao	Shannon	Simpson
AMF colonization %	0.911	0.899	0.866	0.963*	0.940
Fusarium wilt incidence %	-0.859	-0.858	-0.815	-0.958*	-0.885
ERH density	0.931	0.945	0.914	0.999**	0.864
Spore density	0.826	0.795	0.756	0.880	0.949
Total soluble solid (TSS)	0.808	0.777	0.735	0.870	0.939
Soluble sugar (SS)	0.803	0.776	0.732	0.877	0.928
Organic acid (OA)	0.834	0.849	0.802	0.964*	0.818
Vitamin C	0.781	0.828	0.785	0.940	0.655
Soluble protein	0.699	0.690	0.632	0.849	0.814
Nitrate content	0.576	0.581	0.513	0.782	0.688
Fruit N-uptake	-0.829	-0.840	-0.793	-0.958*	-0.829
Fruit P-uptake	0.778	0.732	0.695	0.809	0.951*
Fruit K-uptake	0.794	0.802	0.751	0.935	0.822
Yield	0.812	0.818	0.769	0.943	0.835

*: $P < 0.05$; **: $P < 0.01$.

Table-S6. Correlation analysis of abundant AMF taxa with cucumber fruit development

AMF taxa	TSS	SS	OA	VC	SP	N-content	N-uptake	P-uptake	K-uptake	Yield
Glomus	0.997**	0.954*	0.827	0.985*	0.933	-0.964*	0.968*	0.974*	0.976*	0.982*
Rhizophagous	0.212	0.423	0.555	0.130	0.039	-0.396	0.142	0.327	0.348	0.155
Claroideoglomus	-0.339	-0.273	-0.186	-0.504	-0.627	0.293	-0.308	-0.358	-0.333	-0.334
Funneliformis	0.970*	0.962*	0.860	0.998**	0.980*	-0.971*	0.920	0.986*	0.982*	0.943
Septoglomus	0.713	0.913	0.981*	0.812	0.846	-0.902	0.581	0.892	0.887	0.630
Paraglomus	-0.757	-0.776	-0.709	-0.886	-0.956*	0.787	-0.684	-0.825	-0.808	-0.720
Acaulospora	-0.353	-0.482	-0.529	-0.565	-0.713	0.484	-0.238	-0.521	-0.497	-0.290
Diversispora	0.757	0.776	0.709	0.886	0.956*	-0.787	0.684	0.825	0.808	0.720
Redeckera	-0.832	-0.957*	-0.965*	-0.822	-0.769	0.948	-0.748	-0.922	-0.930	-0.776
Cetraspora	-0.839	-0.629	-0.405	-0.690	-0.546	0.647	-0.912	-0.652	-0.665	-0.885
Gigaspora	0.553	0.755	0.845	0.515	0.446	-0.734	0.460	0.682	0.696	0.486
Ambispora	0.015	-0.036	-0.064	-0.203	-0.378	0.044	0.085	-0.099	-0.070	0.048

* Correlation is significant at the .05 level (2-tailed). ** Correlation is significant at the 0.01 level (2-tailed).
TSS: Total soluble solid; SS: Soluble sugar; OC: Organic acid; VC: Vitamin C; SP: Soluble protein; N-content: Nitrate content

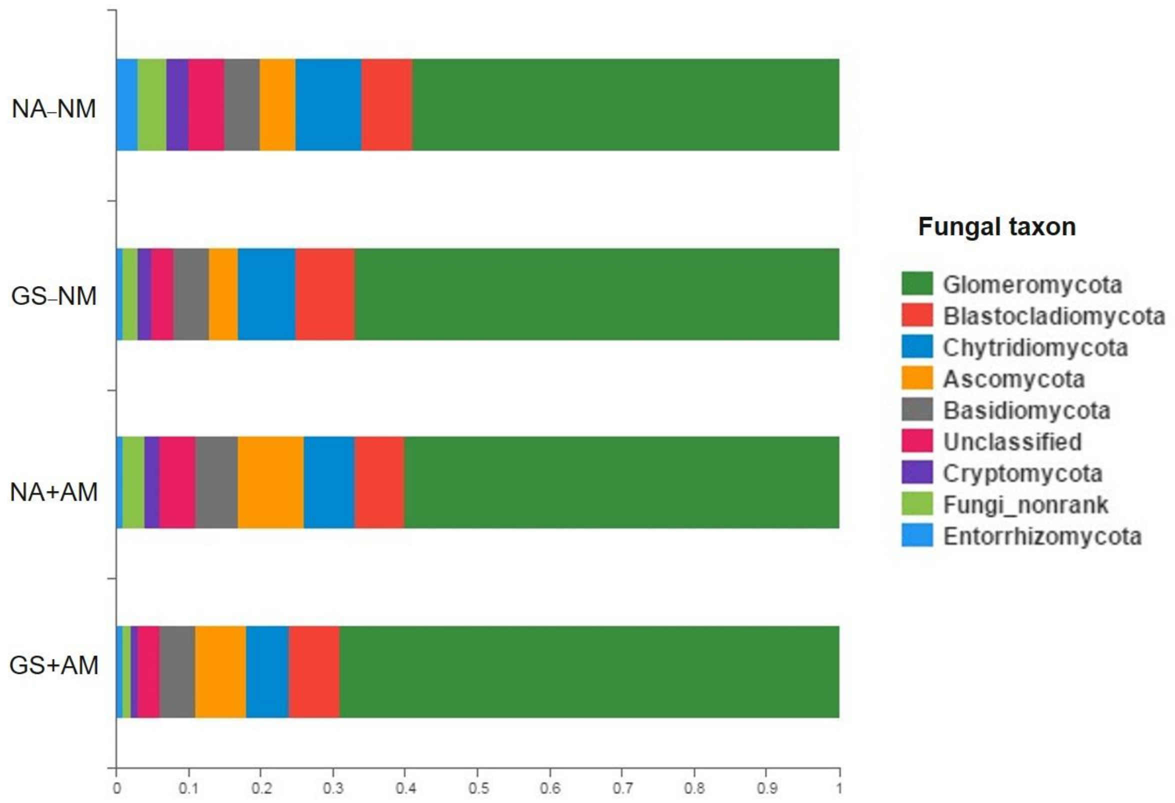

Fig. S1. The proportional relative abundance of in each fungal phylum detected using the primer set AMV4.5NF/AMDGR across all treatment samples. The treatments NA–NM, NA+AM, GS–NM and GS+AM represent the applied soil amendments. Non-amendment and non-mycorrhizal inoculation, non-amended Mycorrhizal Inoculation, Garlic stalk amended with non-mycorrhizal inoculum and Garlic stalk amended with mycorrhizal inoculum, respectively.

Method S1 Calculations for Alpha-diversity indices

In the present study, Chao1 and Ace were used to estimate richness of microbial community. The larger value indicates the higher richness of community in both indices. Shannon's index and Simpson index were used to estimate diversity of microbial community. For Shannon's index, the larger value indicates the higher diversity; while for Simpson's index, the lower value indicates the higher diversity.

Chao1 was expressed by:

$$S_{chao1} = S_{obs} + \frac{n_1(n_1 - 1)}{2(n_2 + 1)}$$

Where S_{chao1} is the calculated numbers of OTUs; S_{obs} is the OTU numbers detected by sequencing; n_1 is the number of "singletons"; n_2 is the number of "doubletons".

Ace was expressed by:

$$S_{ACE} = \begin{cases} S_{abund} + \frac{S_{rare}}{C_{ACE}} + \frac{n_1}{C_{ACE}} \hat{\gamma}^2_{ACE}, & for\ \hat{\gamma}_{ACE} < 0.80 \\ S_{abund} + \frac{S_{rare}}{C_{ACE}} + \frac{n_1}{C_{ACE}} \tilde{\gamma}^2_{ACE}, & for\ \hat{\gamma}_{ACE} \geq 0.80 \end{cases}$$

$$N_{rare} = \sum_{i=1}^{abund} in_i; \quad C_{ACE} = 1 - \frac{n_1}{N_{rare}};$$

$$\hat{\gamma}^2_{ACE} = \max\left[\frac{S_{rare}}{C_{ACE}} \frac{\sum_{i=1}^{abund} i(i-1)n_i}{N_{rare}(N_{rare}-1)} - 1, 0\right];$$

$$\tilde{\gamma}^2_{ACE} = \max\left[\hat{\gamma}^2_{ACE}\left\{1 + \frac{N_{rare}(1 - C_{ACE})\sum_{i=1}^{abund} i(i-1)n_i}{N_{rare}(N_{rare} - C_{ACE})}\right\}, 0\right]$$

Where n_i indicates the number of OTUs which sequences number is i; Srare indicates the number of OTUs which contain "abund" numbers or less then "abund" numbers of sequences; Sabund indicated the number of OTUs which contain more than "abund" numbers of sequences; abund indicate the threshold of average OTU and the defaults is "10".

Shannon’s index was expressed by:

$$H_{shannon} = -\sum_{i=1}^{S_{obs}} \frac{n_i}{N} \ln \frac{n_i}{N}$$

Where S_{obs} indicates the number of detected OTUs; ni indicates the number of sequences contained in the OTU numbered as “i”; N indicates the total number of sequences.

Simpson’s index was expressed by:

$$D_{simpson} = \frac{\sum_{i=1}^{S_{obs}} n_i(n_i - 1)}{N(N-1)}$$

Where S_{obs} indicates the number of detected OTUs; ni indicates the number of sequences contained in the OTU numbered as “i”; N indicates the total number of sequences.
